# Activation of the JAK-STAT Signaling Pathway after *In Vitro* Stimulation with IFNß in Multiple Sclerosis Patients According to the Therapeutic Response to IFNß

**DOI:** 10.1371/journal.pone.0170031

**Published:** 2017-01-19

**Authors:** Isaac Hurtado-Guerrero, Maria Jesús Pinto-Medel, Patricia Urbaneja, Jose Luis Rodriguez- Bada, Antonio León, Miguel Guerrero, Óscar Fernández, Laura Leyva, Begoña Oliver-Martos

**Affiliations:** Unidad de Gestión Clínica de Neurociencias, Instituto de Investigación Biomédica de Málaga (IBIMA), Hospital Regional Universitario de Málaga, Universidad de Málaga, Málaga, Spain; University of Colorado Denver School of Medicine, UNITED STATES

## Abstract

Interferon beta (IFNß) is a common treatment used for multiple sclerosis (MS) which acts through the activation of the JAK-STAT pathway. However, this therapy is not always effective and currently there are no reliable biomarkers to predict therapeutic response. We postulate that the heterogeneity in the response to IFNß therapy could be related to differential activation patterns of the JAK-STAT signaling pathway. Our aim was to evaluate the basal levels and the short term activation of this pathway after IFNß stimulation in untreated and IFNß treated patients, as well as according to therapeutic response. Therefore, cell surface levels of IFNAR subunits (IFNAR1 and IFNAR2) and the activated forms of STAT1 and STAT2 were assessed in peripheral blood mononuclear cells from MS patients by flow cytometry. Basal levels of each of the markers strongly correlated with the expression of the others in untreated patients, but many of these correlations lost significance in treated patients and after short term activation with IFNß. Patients who had undergone IFNß treatment showed higher basal levels of IFNAR1 and pSTAT1, but a reduced response to *in vitro* exposure to IFNß. Conversely, untreated patients, with lower basal levels, showed a greater ability of short term activation of this pathway. Monocytes from responder patients had lower IFNAR1 levels (p = 0.039) and higher IFNAR2 levels (p = 0.035) than non-responders just after IFNß stimulation. A cluster analysis showed that levels of IFNAR1, IFNAR2 and pSTAT1-2 in monocytes grouped 13 out of 19 responder patients with a similar expression pattern, showing an association of this pattern with the phenotype of good response to IFNß (p = 0.013). Our findings suggest that an activation pattern of the IFNß signaling pathway in monocytes could be associated with a clinical phenotype of good response to IFNß treatment and that a differential modulation of the IFNAR subunits in monocytes could be related with treatment effectiveness.

## Introduction

Multiple sclerosis (MS) is a chronic, inflammatory, demyelinating and presumably autoimmune disease of the central nervous system. It is characterized by the presence of multifocal lesions with variable axonal degeneration that result in progressive neurological disability [1]. Interferon beta (IFNß) is a common treatment used for patients with relapsing-onset MS [2]; however there is a large percentage of patients in which the IFNß therapy fails to reduce the relapse rate and the disability progression [3]. Clinicians should identify these non responder patients as early as possible, in order to offer other alternative therapies that are currently available. However, although several efforts have been made, at the moment there is no reliable biomarker to predict the response to IFNß treatment and only neutralizing antibodies (NABs) are considered to be a biomarker of FNß bioactivity that might affect clinical decision making [4].

IFNß exerts its biological activity through interaction with a heterodimeric type I interferon receptor (IFNAR), composed of the IFNAR1 and IFNAR2 subunits [5] and through activation of the JAK-STAT signaling pathway [6]. This interaction results in the phosphorylation of IFNAR1 and IFNAR2 in tyrosine residues by Janus kinases, Tyk2 and JAK1, that subsequently result in the phosphorylation of critical residues of the transcriptional factors of the STAT family, mainly STAT1 (tyrosine 701) and STAT2 (tyrosine 689), leading to the formation of a heterodimer. This heterodimer associates with a third subunit, interferon regulatory factor 9 (IRF9/p48), to form the transcriptional complex IFN-stimulated gene factor 3 (ISGF3), which in turn, translocates to the nucleus to activate genes containing the IFN-stimulated response elements (ISRE) [7–10].

The JAK-STAT pathway is utilized by many other cytokines for signaling, and its activation is critical for the orchestration of immune responses [11]. The dysregulation of this pathway has pathological implications in autoimmune diseases such as rheumatoid arthritis, lupus erythematosus and psoriasis [12–15]. Accordingly, in the last years, therapies targeting the JAK-STAT signaling pathway have emerged for rheumatoid arthritis and other inflammatory diseases [16].

In MS patients, alterations in the JAK-STAT signaling pathway have also been described. The different leukocyte subsets showed a differential activation of STATs in response to systemic injection of IFNß1a or *in vitro* stimulation [17]. Furthermore, the activation of this pathway in MS has been related with different clinical issues. Phosphorylated STAT1 (pSTAT1) has been proposed as a marker of disease activity since an up-regulation of pSTAT1 in peripheral blood mononuclear cells (PBMC) was observed in the active phase of the disease [18]. Moreover, those patients who subsequently became non-responders showed a greater activation in the JAK-STAT signaling pathway before the onset of IFNß therapy, with an elevation of IFNAR1 and pSTAT1 levels in monocytes [19].

The aim of the present study was to assess the levels of some proteins of the JAK-STAT pathway at baseline and after short term activation with IFNß, in untreated and IFNß-treated patients and to determine whether the activation patterns of those proteins discriminated responders from non-responders to IFNß therapy.

## Materials and Methods

### Subjects

This cross-sectional study enrolled forty eight patients with clinically definite relapsing remitting MS [20,21] from the Multiple Sclerosis Unit at Regional University Hospital in Málaga (Spain). Among them, 17 patients were treatment-naive for at least 6 months, and 31 patients were treated with IFNß1a or 1b, for 12–14 months. None of them had received corticosteroids in the three months prior to blood sampling. All the patients were sampled during remissions and, in the case of treated patients after 12–14 months of IFNß therapy, within 10–12 hours post-injection.

The presence of neutralizing antibodies (NABs) against IFNß in serum samples of treated patients was tested by the cytopathic effect test following the WHO recommendations, as previously described [22]. The NABs were tested in the serum samples collected at the same time point as the blood used for isolating PBMC to avoid any possible influence on the activation of the Jak-STAT signaling pathway. Only those patients who were NABs negative were included in the study.

After 12 months of IFNß therapy, patients were classified according to their clinical and MRI activity [23]. Each patient could be positive for relapses, progression or MRI activity. Those patients exhibiting at least one relapse during the first year of therapy were considered positive for relapses; those who showed a progression of disability in the Expanded Disability Status Scale (EDSS) score of at least one point during the first year of therapy (confirmed at 6 months) were considered positive for progression; finally, those developing three or more active lesions (either new or enlarging T2 lesions compared with baseline MRI scan or gadolinium-enhancing lesions) on the MRI performed after 1 year of therapy were classified as positive for MRI activity.

Patients were considered non-responders according to the occurrence of two or three positive variables during the first year of therapy, otherwise they were considered responders.

The samples were provided by the Biobank of the Andalusian Public Health System. All patients participating in the study gave their written informed consent and protocols were approved by institutional ethical committees (CEI Málaga Nordeste).

The demographic and clinical characteristics of the subjects are summarized in Table 1.

**Table 1:** Demographic and clinical characteristics of multiple sclerosis patients.

	Untreated	IFNß Treated	p-Value (a)	Responders	Non-responders	p-Value (b)
Number of subjects	17	31		19	12	
Female/male	10/7	19/12	n.s.	12/7	6/6	n.s.
Age (years)	37 ± 11	36 ± 10	n.s.	36 ± 10	35 ± 11	n.s.
MS duration (years)	6.5 ± 5.4	6.9 ± 5.4	n.s.	6.4 ± 5.6	7.7 ± 5.2	n.s.
EDSS at baseline	1 (0–3.75)	1 (0–1.5)	n.s.	0 (0–1)	1 (0–2.5)	n.s.
EDSS after a year of therapy	-	1 (0–1.5)		0 (0–1)	1.75 (1–2.875)	0.003
Number of relapses before treatment onset	1 (0–1.5)	1 (0–1)	n.s.	1(1–1)	1(0–2)	n.s.
Number of relapses in the first year ofof therapy	-	0 (0–1)		0(0–0)	1(1–1)	9 x10^-6^
Number of patients with MRI activity before interferon onset	-	-		6/19	10/12	<0.005
Number of patients with MRI activity after 1 year of IFNß therapy	-	-		1/19	9/12	<0.00005

Quantitative data are presented as mean ± standard deviation (age and MS duration) or as median (inter-quartile range) for EDDS and number of relapses.

^(a)^ P-values obtained between untreated and treated patients by chi-square test (gender), T-test (age, duration) or Mann-Whitney test (EDSS and number of relapses).

^(b)^ P-values obtained between responder and non responder patients by chi-square test (gender, MRI activity), T-test (age, duration) or Mann-Whitney test (EDSS and number of relapses).

*n*.*s* non significant, *MS* multiple sclerosis, *EDSS* expanded disability status scale, *IFNß* interferon beta.

### Sample collection

Fresh lithium heparinised blood was obtained by venipuncture from clinically stable MS patients. PBMC were isolated using a ficoll-hypaque gradient, as described in the supplier's protocol (ICN Biomedicals Inc., OH, USA). After that, cells were cryopreserved in RPMI-1640 medium (BioWhittaker), 40% heat-inactivated fetal calf serum (FBS) (BioWhittaker) and 10% DMSO (Sigma), until use.

### IFNß stimulation and flow cytometry

A panel of four markers was selected including the two subunits of IFNAR (IFNAR1 and IFNAR2) and the activated forms of STAT (pSTAT1 and pSTAT2), to cluster patients based on signaling profiles.

PBMC from untreated and treated MS patients were thawed and suspended in pre-warmed RPMI-1640 medium (BioWhittaker), supplemented with 2 mM L-glutamine (ICN Biomedicals), 20% FBS (BioWhittaker) and 0.032 mg/ml gentamicin (Normon). PBMC were washed by centrifugation, re-suspended in the same medium without FBS (1 million /ml) and incubated at 37°C for 90 min, in order to obtain the lowest level of activation.

We first analyzed early kinetics of STAT phosphorylation induced by a single dose of 1000 IU/ml IFNß1a (Avonex, Biogen, Inc.) in Jurkat cells. Then, the preliminary experiments with PBMC from MS patients showed that within 30 min of stimulation, phosphorylation of STAT1 and STAT2 was close to maximum levels, so we chose this time and dose for cell stimulation in the study (data not shown).

Cells from each subject were stimulated either with 1000 IU/ml IFNß1a to allow the signal transduction and the phosphorylation of the proteins or with RPMI-1640 medium without FBS during 30 minutes at 37°C (unstimulated cells considered as basal levels of activation).

Following stimulation, the cells were fixed with Cytofix (BD Biosciences) at 37°C for 10 min, washed twice with Perm/Wash Buffer (BD Biosciences) and permeabilized with PermBuffer III (BD Biosciences) at 4°C for 20 min. After two additional washes, the cells were stained for 30 min in the dark, at room temperature with fluorescein isothiocyanate, phycoerythrin, phycoerythrin-cyanine, peridin chlorophyll protein, Alexa Fluor-488 and allophycocyanin labelled specific monoclonal antibodies (MAb) for the following molecules: IFNAR1(R&D, FAB245F), IFNAR2 (PBL 21385–3), phospho-STAT1 (Y^701^) (BD Biosciences 612596), phospho-STAT2 (Y^689^) (R&D IC2890F), CD3 (BD Biosciences 345766), CD8 (BD Biosciences 560917) and CD14 (BD Biosciences 555399) S1 Table. Previously, the antibodies were titrated for optimal separation and staining. Four or five colour stainings with different combinations of MAb were performed in order to evaluate the expression of the IFNAR subunits and the activation of STATs in the different cell populations. Isotype-matched controls were used to verify the staining specificity of the antibodies.

**Fig 1:**
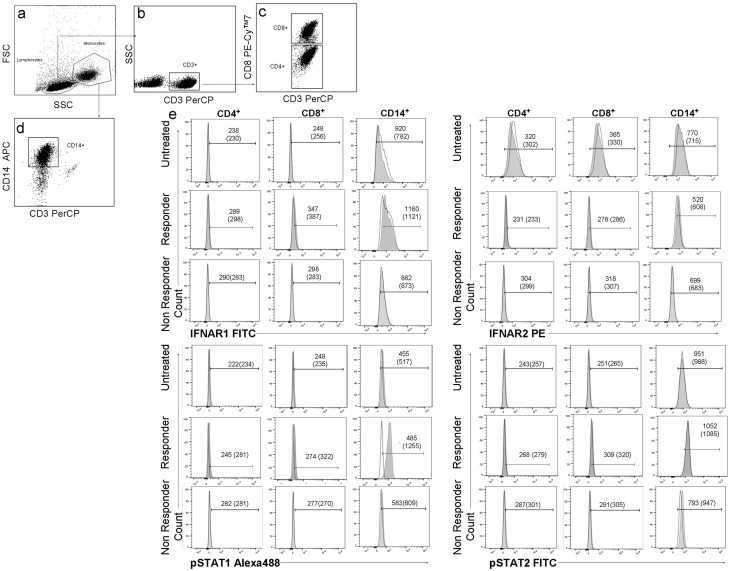
Gating strategy to determine IFNAR1, IFNAR2, pSTAT1 and pSTAT2 expression on different lymphocyte subsets. First, based on the combination of FSC (size) and SSC (granularity) attributes of the acquired events, lymphocyte and monocyte gates were selected (a). T lymphocytes were identified by gating on CD3+cells (b), then they were transferred to a new dot plot and were analyzed by a specific antibody against CD8. T CD8+ lymphocytes were identified as CD3+ and CD8+ cells and T CD4+ lymphocytes as CD3+ and CD8- cells (c). Monocytes were identified as CD14+ and CD3- (d). Overlay histograms from a representative patient of each study group that depict the MFI of unstimulated (open histograms) and IFNß-stimulated (1000UI/mL, 30 min) (filled histograms) cells expressing IFNAR1, IFNAR2, pSTAT1 and pSTAT2 in CD4+, CD8+ T lymphocytes and monocytes. The numbers next to each of the histograms indicate the MFI values for unstimulated and stimulated cells (in brackets) (e). *FSC*: forward scatter, *SSC*: side scatter, *FITC*: fluorescein isothiocyanate, *PE*: phycoerythrin, *PE-Cy7*: phycoerythrin-cyanine, *PerCP*: peridin chlorophyll protein, *Alexa488*: Alexa Fluor-488, *APC*: allophycocyanin.

Cells were washed and acquired in a FACSCanto II^™^ flow cytometer (BD Biosciences) using the FACSDiva software (BD Biosciences). At least 50,000 events were acquired from each sample. The gating strategy is shown in Fig 1. The expression of IFNAR1, IFNAR2, pSTAT1 and pSTAT2 was determined in unstimulated and IFNß-stimulated CD4^+^, CD8^+^ T cells and monocytes, and the data were analyzed as geometric mean fluorescent intensities (MFI) for each marker within each cell subpopulation.

### Statistical analysis

Expression of each variable was tested for distribution using the Kolmogorov–Smirnov test. Distribution of the quantitative variables following a normal distribution (age and MS duration) in the patient subgroups were analyzed by the T test. In the case of variables that did not follow a normal distribution (EDSS and number of relapses), non-parametric tests were applied.

Basal levels of expression in unstimulated cells were analyzed as MFI, and the Mann-Whitney U test was used for the comparisons between two groups. Changes in IFNAR1, IFNAR2, pSTAT1 and pSTAT2 after *in vitro* stimulation with IFNß were analyzed as the following ratio: MFI of stimulated cells / MFI of unstimulated cells (MFI_s_/MFI_us_) using the Mann-Whitney U test. For the heat map representation, log_2_ was applied to this ratio [24]. Statistical significance was set at p ≤0.05. The correlation between the expression of the different markers in MS patients was assessed using the Pearson correlation coefficient.

Four parameters (IFNAR1, IFNAR2, pSTAT1 and pSTAT2) were included as variables in an unsupervised average linkage hierarchical clustering, executed with Genesis software [25] to search for a specific pattern related to therapeutic response to IFNß. After that, the variable "presence or absence of the pattern identified by the clustering" was associated with the response to IFNß therapy by the chi-square test.

## Results

### Correlations of protein levels

#### Baseline correlation

**Fig 2:**
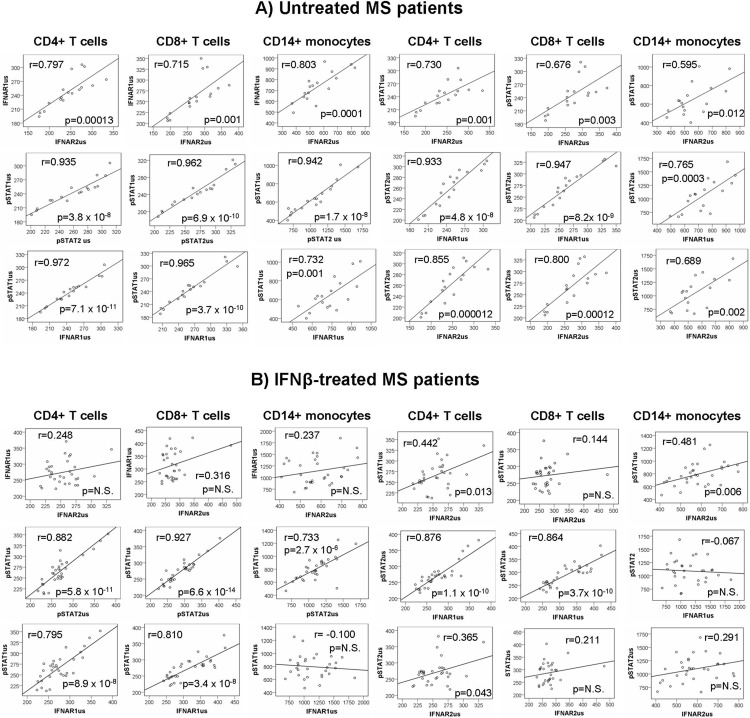
Correlations between the levels of expression of the different markers in the three unstimulated cell subsets (CD4+ T cells, CD8+ T cells, CD14+ monocytes) A) In untreated MS patients; B) In IFNß-treated MS patients. The correlation between the MFI_us_ of the different markers was assessed using the Pearson correlation coefficient. MFI_us_: mean fluorescence intensity in unstimulated cells

Untreated patients showed a strong positive correlation between the expression levels of each of the markers with the levels of the other three markers in the three unstimulated cell subsets (Fig 2A). However, in IFNß treated patients, IFNAR1 levels did not correlate with IFNAR2 levels in any of the cell subsets. Additionally, IFNAR1 levels did not correlate with STAT1 or STAT2 levels in monocytes, and IFNAR2 levels did not correlate with STAT1 in CD8+ T cells or with STAT2 levels in CD8+ T cells and monocytes, as observed in Fig 2B.

#### Correlation after short-term in vitro stimulation with IFNß

**Fig 3:**
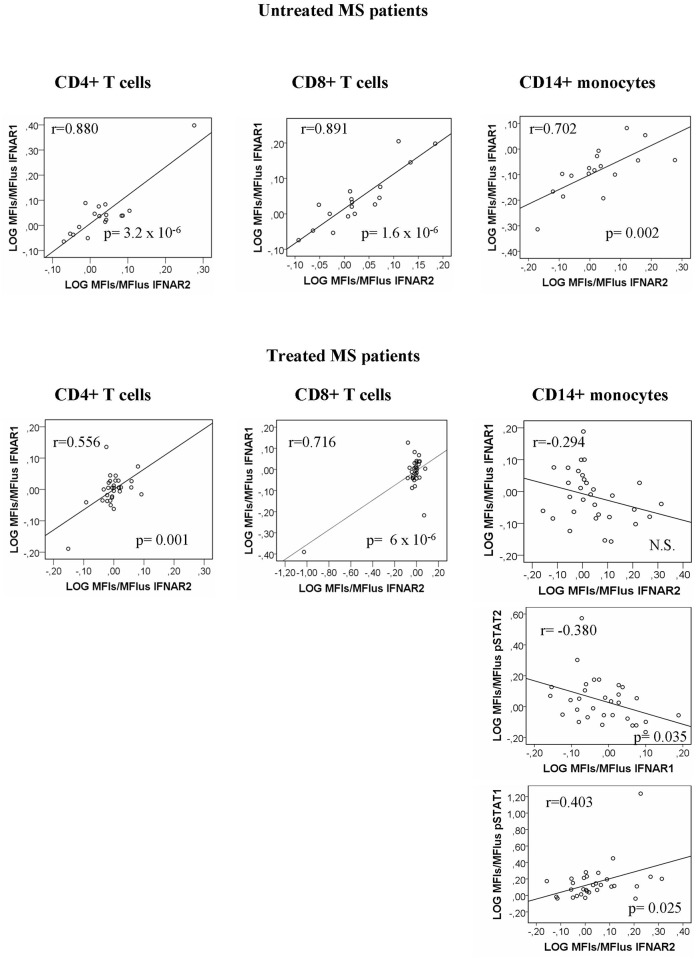
Correlations between levels of expression of the different markers in the three stimulated cell subsets (CD4+ T cells, CD8+ T cells, CD14+ monocytes) in untreated and IFNß-treated MS patients. The conditions of IFNß stimulation were 1000UI/mL, 30 min. The correlation between the log_2_ [MFI_s_/ MFI_us_] of the different markers was assessed using the Pearson correlation coefficient. MFI_s_: mean fluorescence intensity in IFNß-stimulated cells MFI_us_: mean fluorescence intensity in unstimulated cells

Correlations between the expression levels of each of the markers with the levels of the other three markers in the three cell subsets were lost after *in vitro* activation with IFNß. In untreated patients, only IFNAR1 expression correlated with IFNAR2 expression in the 3 cell subsets. This correlation was replicated in the treated patients in the T cell subsets, but was lost in the stimulated monocytes, in which the only correlations observed were between IFNAR1-STAT2 levels and IFNAR2-STAT1 levels (Fig 3).

### JAK-STAT signaling pathway in unstimulated cells and after short term IFNß stimulation. Comparisons between untreated and treated patients

#### Baseline expression

Before evaluating the effects of IFNß stimulation, the expression of the steady-state levels of each of the markers of the JAK-STAT signaling pathway was assessed in three cellular subsets (CD4+ T cells, CD8+ T cells, and CD14+ monocytes) in untreated and treated MS patients. Among all the subsets, the monocytes showed the highest MFI for all the markers in unstimulated conditions, as shown in Table 2.

**Table 2:** Mean Fluorescence Intensity of IFNAR1, IFNAR2, pSTAT1 and pSTAT2 in unstimulated cells. Comparison between IFNß treated and untreated MS patients.

MFI IN UNSTIMULATED CELLS				
		Non-treated	Treated	p
CD4	IFNAR1	247.65 ± 33.52	274.9 ± 37.79	0,019
	IFNAR2	243.82 ± 43.61	254.9 ± 25.10	NS
	pSTAT1	243.12 ± 28.74	266.52 ± 33.81	0,020
	pSTAT2	259.88 ± 35.28	270.71 ± 38.20	NS
CD8	IFNAR1	262.23 ± 42.52	315.97 ± 54.96	0,001
	IFNAR2	270.41 ± 53.31	277.16 ± 45.08	NS
	pSTAT1	245.76 ± 37.75	273.58 ± 35.16	0,026
	pSTAT2	267.88 ± 40.08	288.16 ± 41.94	NS
CD14	IFNAR1	735.36 ± 136.79	1143.81 ± 290.34	<0,0001
	IFNAR2	556.59 ± 130.45	583.29 ± 100.18	NS
	pSTAT1	648.23 ± 173.80	794.48 ± 176.49	0,008
	pSTAT2	1061.18 ± 298.78	1090.29 ± 232.95	NS

Data are expressed as mean values ± standard deviation

**Fig 4:**
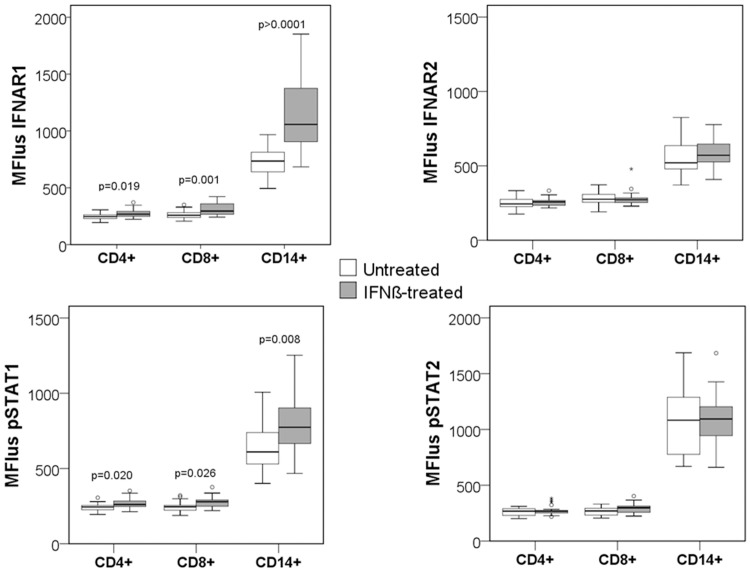
Differences in the expression of IFNAR1, IFNAR2, pSTAT1 and pSTAT2 in unstimulated cells between untreated and treated patients. MFI_us_ of IFNAR1, IFNAR2, pSTAT1 and pSTAT2 in CD4+, CD8+ and CD14+ subsets from untreated and treated patients. The Mann-Whitney U test was used for the comparison between two groups.

The differences in the basal levels between untreated and treated patients were assessed by comparing MFI in unstimulated cells from both groups of patients. IFNß treated patients showed significantly higher MFI_us_ of IFNAR1 and pSTAT1 in the CD4+ T cells (p = 0.019; p = 0.020), CD8+ T cells (p = 0.001; p = 0.026), and monocytes (p<0.0001; p = 0.008) than untreated patients, as observed in Table 2 and Fig 4.

To evaluate the different profiles of the JAK-STAT signaling pathway in unstimulated cells, data of each study group were represented in a heat map. To normalize the data, the average per group of the MFI in unstimulated cells(MFI_us mean group_) was divided by the average of the MFI from all the patients (MFI_us mean all_) for a specific marker, in each of the unstimulated cell subsets, and then, the ratios (MFI_us mean group_ / MFI_us mean all_) for IFNAR1, IFNAR2, pSTAT1, pSTAT2 were log_2_ transformed.

**Fig 5:**
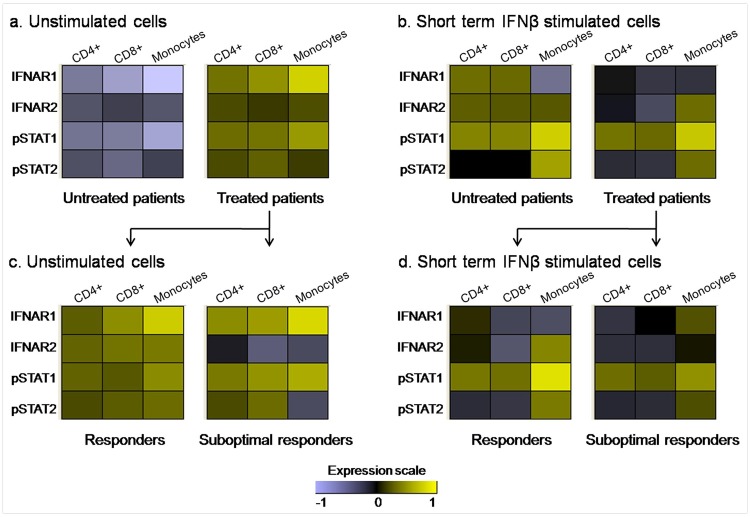
Heat maps showing different profiles of the JAK-STAT signaling pathway in unstimulated cells and after short term IFNß stimulation. The logarithmic transformation of MFI of IFNAR1, IFNAR2, pSTAT1 and pSTAT2 has been represented for each sub-population in: a) Untreated and treated patients in unstimulated conditions log_2_ (MFI_us mean group_ / MFI_us mean all_). b) Untreated and treated patients after short term IFNß stimulation (1000UI/mL, 30 min) log_2_ (MFI_s_/MFI_us_). c) Responders and sub-optimal responders in unstimulated conditions log_2_ (MFI_us mean group_ / MFI_us mean all_). d) Responders and sub-optimal responders after short term IFNß stimulation (1000UI/mL, 30 min) log_2_ (MFI_s_/MFI_us_). In unstimulated cell heat maps (Fig a and c), the data were normalized as follows: the average per group of the MFI in unstimulated cells (MFI_us mean group_) was divided by the average of MFI (MFI_us mean all_) from all the patients, for a specific marker. Then, the ratios (MFI_us mean group_ / MFI_us mean all_) were log2 transformed. In the condition of short term IFNß stimulation cells (Fig b and d), for each patient, the data were normalized by the MFI of unstimulated cells (MFIs/MFIus). For the representation in the heat maps, the average of the ratio of MFIs/MFIus in each study group was calculated and then was log2 transformed. Unchanged proteins are displayed in black, up-regulated proteins are displayed in yellow while the down-regulated proteins are depicted in violet.

These values were represented graphically by colouring log ratios of 0 in black (unchanged proteins), increasingly positive log ratios with yellows of increasing intensity and increasingly negative log ratios with violets of increasing intensity. Long term IFNß-treated patients showed a higher activation pattern in unstimulated conditions than untreated patients, as can be observed in the heat map (Fig 5a).

#### Expression after short-term in vitro stimulation with IFNß

**Fig 6:**
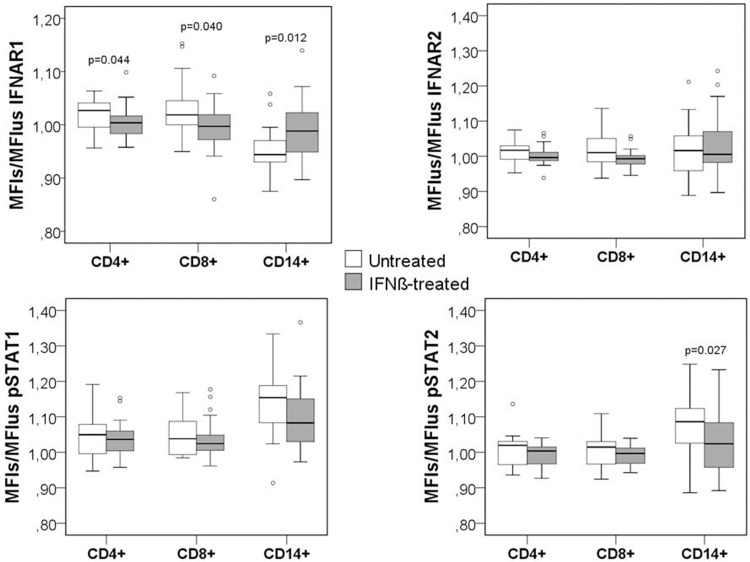
Differences in the expression of IFNAR1, IFNAR2, pSTAT1 and pSTAT2 after short term IFNß stimulation between untreated and IFNß-treated MS patients. Expression is represented as a ratio of the mean fluorescence intensity in stimulated cells (MFI_s_) between the MFI of unstimulated cells (MFI_us_) for IFNAR1, IFNAR2, pSTAT1 and pSTAT2 in CD4+, CD8+ and CD14+ subsets from untreated and treated patients. The conditions of IFNß stimulation were 1000UI/mL, 30 min. The Mann-Whitney U test was used for the comparison between the two groups.

The differences of the *in vitro* stimulation with IFNß between untreated and IFNß treated patients were analyzed as the ratio (MFI_s_/MFI_us_) for IFNAR1, IFNAR2, pSTAT1, pSTAT2 in each cell subset. This ratio provided us with information about the activation state of the cells with short term stimulation with IFNß and, therefore, indirectly, about the functionality of the IFNß signaling pathway. With IFNß stimulation, monocytes from untreated patients showed lower IFNAR1 expression (p = 0.012) and higher STAT2 expression (p = 0.027) than those from treated patients, but showed no differences in IFNAR2 and pSTAT1 levels between both groups. Conversely, in both T cell subsets, *in vitro* stimulation with IFNß slightly increased IFNAR1 levels to a higher extent in untreated patients than in those patients previously treated with IFNß (p = 0.044 and p = 0.040, respectively in CD4+ and CD8+ T cells), as shown in Fig 6.

To look for different profiles of activation of the JAK-STAT signaling pathway after *in vitro* IFNß stimulation, the averages of MFI_s_/MFI_us_ for IFNAR1, IFNAR2, pSTAT1, pSTAT2 of each study group were log_2_ transformed and represented in a heat map, as explained before. Overall, the heat map revealed that untreated patients showed a higher activation pattern after stimulation with IFNß than patients previously treated with prolonged systemic IFNß therapy (Fig 5b).

### JAK-STAT signaling pathway in responder and sub-optimal responder patients

#### Baseline expression

Basal levels of the JAK-STAT signaling pathway markers in treated patients according to the therapeutic response to IFNß were assessed in unstimulated cells. No significant differences in the MFIus of IFNAR1, IFNAR2, pSTAT1 and pSTAT2 in the three cell subsets were observed between responder and sub-optimal responder patients, as shown in Table 3. As mentioned before, the highest MFI for all the markers were observed in the monocyte subset.

**Table 3:** Mean Fluorescence Intensity of IFNAR1, IFNAR2, pSTAT1 and pSTAT2 in unstimulated cells (MFI_us_). Comparison between responders and sub-optimal responders to IFNß therapy.

MFI IN UNSTIMULATED CELLS				
		Responders	Non responders	p
CD4	IFNAR1	271.32 ± 41.39	280.58 ± 32.15	N.S.
	IFNAR2	257.84 ± 25.64	250.25 ± 24.58	N.S.
	pSTAT1	264.95 ± 36.31	269 ± 30.80	N.S.
	pSTAT2	270.68 ± 43.61	270.75 ± 29.46	N.S.
CD8	IFNAR1	314.21 ± 61.94	318.75 ± 44.10	N.S.
	IFNAR2	285.05 ± 51.87	264.67 ± 29.37	N.S.
	pSTAT1	269 ± 39.80	280.83 ± 26.18	N.S.
	pSTAT2	287.11 ± 48.55	289.83 ± 30.58	N.S.
CD14	IFNAR1	1136.58 ± 281.91	1155.25 ± 315.64	N.S.
	IFNAR2	597.37 ± 71.61	561 ± 134.52	N.S.
	pSTAT1	784.79 ± 159.39	809.83 ± 207.25	N.S.
	pSTAT2	1113.26 ± 229.07	1053.92 ± 244.51	N.S.

Data are expressed as mean values ± standard deviation

At baseline, levels of IFNAR1 correlated with levels of STAT1 and STAT2 in both T cell subsets, both in responders and non-responders. Additionally, STAT1 expression correlated with STAT2 expression in the three cell subsets in both groups, as shown in Table 4.

**Table 4:** Significant correlations between Mean Fluorescence Intensities of IFNAR1, IFNAR2, pSTAT1 and pSTAT2 in unstimulated cells (MFI_us_). Comparison between responders and sub-optimal responders to IFNß therapy.

Correlations	Response to IFNß therapy	CD4+		CD8+		CD14+	
		r	p	r	p	r	p
IFNAR1-STAT1	R	0.850	4 x 10^−8^	0.828	1.2 x 10^−5^		
	NR	0.662	0.019	0.772	0.003		
IFNAR1-pSTAT2	R	0.926	1.2 x 10^−8^	0.893	2.7 x 10^−7^		
	NR	0.752	0.005	0.765	0.004		
pSTAT1-pSTAT2	R	0.933	5.5 x 10^−9^	0.960	7.1 x 10^−11^	0.652	0.002
	NR	0.757	0.004	0.838	0.001	0.873	0.0002

R: responders; NR: non-responders; r: Pearson correlation coefficient

To construct the heat map in unstimulated cells, the data of each marker were normalized as follows: log_2_ (MFI_us mean group_ / MFI_us mean all_). Although some differences can be seen between both groups, they did not reach statistical significance (Fig 5c).

#### Expression after short-term in vitro stimulation with IFNß

**Fig 7:**
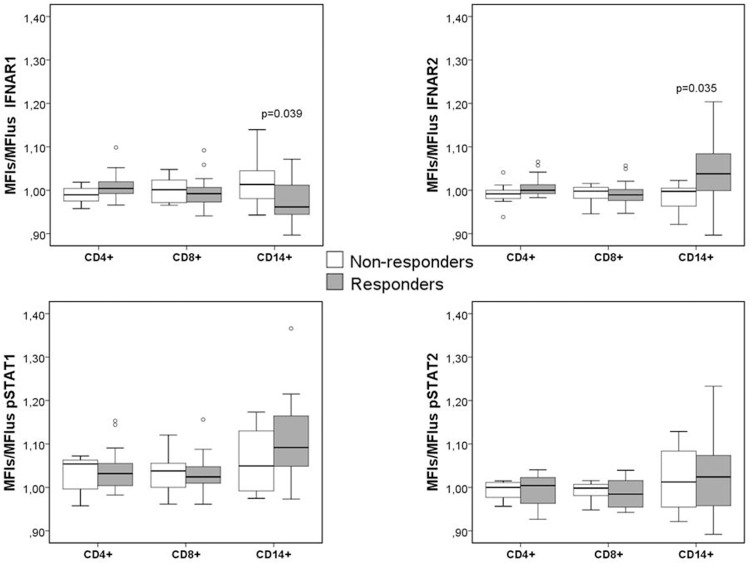
Differences in the expression of IFNAR1, IFNAR2, pSTAT1 and pSTAT2 after short term IFNß stimulation between responders and sub-optimal responders. Expression is represented as the ratio of the mean fluorescence intensity in stimulated cells (MFI_s_) between the MFI of unstimulated cells (MFI_us_) of IFNAR1, IFNAR2, pSTAT1 and pSTAT2 in CD4+, CD8+ and CD14+ subsets from responder and non-responder patients. The conditions of IFNß stimulation were 1000UI/mL, 30 min. The Mann-Whitney U test was used for the comparison between the two groups.

The differences in the activation of the signaling cascade after stimulation with IFNß between responders and sub-optimal responders were analyzed as the ratio (MFI_s_/MFI_us_) for each of the four markers, S2 Table. Monocytes from responder patients showed slightly lower IFNAR1 levels (p = 0.039) and higher IFNAR2 levels (p = 0.035) than monocytes from non-responders (Fig 7). However, differences between responders and non responders in the ability to activate STAT proteins (assessed by tyrosine phosphorilation) after IFNß stimulation were subtle and did not reach statistical significance.

**Fig 8:**
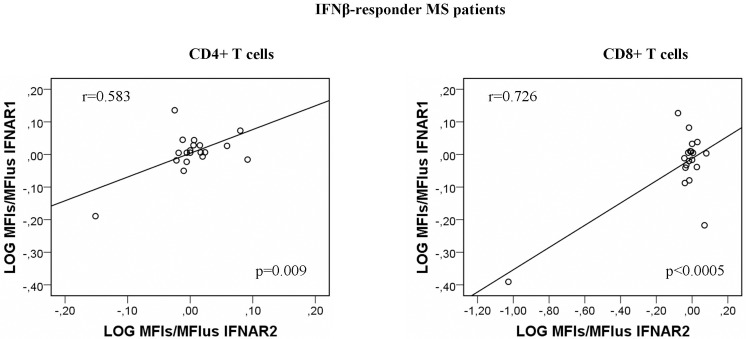
Correlation of IFNAR1 log_2_ (MFI_s_/MFI_us_) and IFNAR2 log_2_ (MFI_s_/MFI_us_) expression in T cells from responder patients.

After short term *in vitro* activation with IFNß, the changes in the levels of IFNAR1 and IFNAR2 correlated significantly in CD4+ (r = 0.583; p = 0.009) and CD8+ T cells from responders exclusively (r = 0.726; p = 0.00043), a correlation that was not found in monocytes (Fig 8).

#### Different patterns of JAK-STAT signaling pathway after in vitro stimulation according to therapeutic response

**Fig 9:**
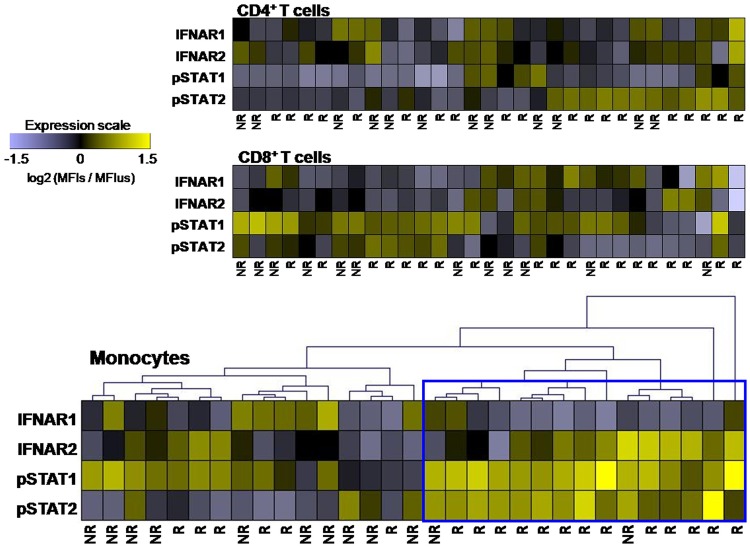
Individual activation pattern of JAK-STAT signaling pathway after IFNß in vitro stimulation related with the responsiveness to IFNß therapy. The log_2_ (MFI_s_/MFI_us_) of IFNAR1, IFNAR2, pSTAT1 and pSTAT2 of each treated patient were represented in a heat map for each of the cellular sub-populations. Unchanged proteins are displayed in black, over-expressed proteins are displayed in yellow, while down-regulated proteins are shown in violet. In monocytes, a non- supervised average linkage hierarchical clustering grouped 13 out of 19 of responder patients with a similar expression pattern (blue square). *MFI*_*s*_ mean fluorescence intensity of stimulated cells, *MFI*_*us*_ mean fluorescence intensity of unstimulated cells, *NR* non-responders to IFNß therapy, *R* responders to IFNß therapy.

As in the other conditions, the average of log_2_ (MFI_s_/MFI_us_) of responders and non-responders was represented in a heat map (Fig 5d), where some differences could be observed. In order to identify the different functional patterns in the IFNß signaling pathway related to the therapeutic response to IFNß, the log_2_ (MFI_s_/MFI_us_) of each patient was further represented in a heat map (Fig 9). The unsupervised average linkage hierarchical clustering showed that the levels of IFNAR1, IFNAR2, pSTAT1 and pSTAT2 in monocytes were able to group 13 out of 19 (68%) responders with a similar expression pattern. This "responder" pattern in monocytes was characterized by a decrease in IFNAR1 levels, and a simultaneous increase in IFNAR2, pSTAT1 and pSTAT2 after *in vitro* stimulation with IFNß. The chi-square test showed an association between the presence of this pattern and the clinical phenotype of good therapeutic response to IFNß (p = 0.013). However, this pattern related with the therapeutic response to IFNß could not be identified in CD4^+^ or CD8^+^ T cell sub-populations.

## Discussion

The mechanism of IFNß therapy in relapsing-remitting multiple sclerosis is not completely understood, however it is well known that its action starts with the interaction with IFNAR and the activation of the JAK and STAT proteins[26]. The evaluation of the cell surface receptor and the phosphoproteins just after IFNß1a stimulation allows us to characterise the short term activation of the JAK-STAT signaling pathway. Our hypothesis was that a differential activation of this pathway could help to explain differences in responsiveness to IFNß therapy and that patients could be grouped based on signaling profiles.

First of all, the signaling pathway was characterized in untreated and IFNß-treated patients, to evaluate the effect of the prolonged systemic IFNß therapy. Physiological expression levels of IFNAR1 and IFNAR2 are very low [27] and, as other low copy-number proteins, IFNAR1 and IFNAR2 show a large variance in the receptor concentrations of individual cells [28]. Strikingly, in our cohort, basal levels of each of the markers correlated strongly with the expression of the others in untreated patients, but many of these correlations lost significance in previously treated patients, so systemic therapy with IFNß is influencing the basal expression of these markers. Accordingly, it has been described that type I IFN binding to their receptor induces IFNAR1 internalization and degradation via lysosomal receptor proteolysis [29–33], while IFNAR2 expression displays a considerably slower basal turnover [33].

Under unstimulated conditions, levels of IFNAR1 and pSTAT1 were significantly higher in IFNß-treated patients than in untreated patients in the three sub-populations. However, after short term stimulation with IFNß, monocytes from untreated patients had a more prominent response to the stimulus than those from treated patients, showing a higher decrease in the expression of IFNAR1 and a higher activation of pSTAT2. Accordingly, monocytes and T cells from patients with prolonged IFNß treatment showed higher basal levels of IFNAR1 and pSTAT1 but showed a reduced response to *in vitro* exposure to IFNß. Conversely, untreated patients, with a lower activation state under unstimulated conditions, showed a higher ability of short term activation of the pathway after *in vitro* stimulus with IFNß. These results suggest a de-sensitization to the IFNß stimulus in previously treated patients as a consequence of the continued exposure to IFNß treatment [34, 35]. Otherwise, STAT-1, and -2 were not fully constitutively activated in T cells and monocytes, as *in vitro* stimulation with IFNß increased their activation in both untreated and treated patients, although the ability to respond to this cytokine by activating pSTATs was slightly reduced in previously treated patients. Continuous systemic therapy with IFNß may diminish the capacity of these cells to respond to this cytokine through these transduction factors.

Among all the analyzed cell subsets, monocytes showed a higher response to *in vitro* stimulation with IFNß than CD4^+^ and CD8^+^ T cells, so IFNß stimulation seems to trigger cell type specific responses, as has been previously described in MS [17].

Regarding the therapeutic response to IFNß, it has been previously described that before the onset of IFNß therapy, those patients who subsequently became non responders showed higher IFNAR1 and pSTAT1 levels in monocytes than those who responded satisfactorily to this therapy [19], but there is a lack of JAK-STAT pathway data concerning patients undergoing IFNß treatment, as in this study.

We have shown that responders and non-responders showed no differences in the expression of any of the markers under steady-state conditions. However, responders are the group that better reflect the modulation of biological responsiveness to *in vitro* IFNß stimulation. Monocytes from responder patients underwent a rapid down-modulation of the cell surface IFNAR1 and an up-regulation of the IFNAR2 subunit. This observation suggests that, in non-responders a more important de-sensitization of the JAK-STAT pathway could occur as a consequence of the prolonged systemic treatment [34]. This could be a cause that justifies the lack of response with an important biological rationale, that should be investigated in a greater cohort of patients.

We thought that it would be interesting to address whether a differential modulation of the subunits occurs according to the therapeutic response, as the modulation of IFNAR1 and IFNAR2 levels is one of the main regulatory mechanisms of the duration and potency of cell responsiveness to IFNß. Several studies have demonstrated that IFNß induces endocytosis and degradation of IFNAR1 to regulate the cell signaling [36–38] en just 30 minutes after IFNß stimulation, a down-regulation of IFNAR1 has been described in some cell lines, while IFNAR2 expression displayed a considerably slower basal turnover and the extent of its down-regulation diminished as IFNAR1 expression decreased in cells [33]. Additionally, although IFNAR2 is the binding subunit, IFNAR1 can also interact with IFNß and the complex is able to transmit signals to induce genes independently of IFNAR2 [39], a relevant fact that could explain differences in the response to IFNß treatment.

In our study, after *in vitro* stimulation with IFNß, the cell surface expression of IFNAR1 decreased while that of IFNAR2 increased in monocytes from responders. These changes suggest that, in responders, a differential regulation of the two receptor subunits is possible and the increase in IFNAR2 surface expression may compensate the well known IFNAR1 down-regulation induced by continuous exposure to IFNß, in an attempt to maintain the activation of the JAK-STAT signaling pathway that will result in transcriptional activation or repression of interferon-regulated genes.

The changes of the cell surface receptor and the phospho-proteins just after IFNß *in vitro* stimulation were used to address whether the patients could be grouped based on the activation of the JAK-STAT signaling pathway, using an unsupervised linkage hierarchical clustering. It was possible to identify a pattern in monocytes that was present in 68.4% of responder patients based on the similarity of their cell surface receptor levels and the expression of phospho-STAT proteins. This pattern, significantly associated with the phenotype of good responsiveness to IFNß treatment, was characterised by a decrease in IFNAR1 levels and an increase in IFNAR2, pSTAT1 and pSTAT2 levels upon stimulation with IFNß. Only two non-responders were also grouped within this pattern, so that the lack of response in these patients seems not to be directly related with the JAK-STAT signaling pathway, nor with the presence of NABs, as all the patients included in the study were NABs negative. Other factors that have not be considered in the study, such as the lack of compliance could explain this lack of response.

As JAK-STAT signaling alone is not enough to explain all the biological effects of type I IFNs, and the molecular mechanisms of the therapeutic response to IFNß are not yet completely understood, it would be interesting to study other alternative kinases or transcription factors, as well as the inclusion of other downstream markers in the IFNß signalling pathway that could explain why some responder patients did not cluster within the profile of good response and that would have strengthened the study.

Due to the complexity of MS pathology and the heterogeneity in the response to therapy, it might be very difficult to establish a single response biomarker. The approach in this study has allowed us to associate a clinical phenotype of good responsiveness to IFNß treatment with a functional pattern of the IFNß signaling pathway in monocytes, but further investigation is required to elucidate completely the role of the JAK-STAT signaling pathway in the responsiveness to IFNß therapy.

## Supporting Information

S1 Table Detailed information of the antibodies used in flow cytometry. (DOC)

S2 Table Data set responder and non-responder patients. (XLS)
