# Supplementary material for: Activation of the JAK-STAT Signaling Pathway after In Vitro Stimulation with IFNß in Multiple Sclerosis Patients According to the Therapeutic Response to IFNß
Source: PLoS One. 2017 Jan 19;12(1):e0170031. doi: 10.1371/journal.pone.0170031 (PMC5245989; doi:10.1371/journal.pone.0170031)
Supplement: S1 Table — (DOC) [file pone.0170031.s001.doc]

| **Name** | **Monoclonal or Polyclonal** | **Host species** | **Commercial supplier** | **Catalogue Number** | **Antigen used to raise the antibody** | **Final antibody dilution** | **Label** |
| --- | --- | --- | --- | --- | --- | --- | --- |
| CD3 PerCP | Monoclonal | Mouse | BD Biosciences | 345766 | Human thymocytes | 1:35 | Peridin Chlorophyll Protein (PerCP) |
| PE-Cy™7 Mouse Anti-Human CD8 | Monoclonal | Mouse | BD Biosciences | 557746 | Human CD8a | 1:100 | Phycoerythrin-Cyanine Dye(PE-Cy™7) |
| APC Mouse Anti-Human CD14 | Monoclonal | Mouse | BD Biosciences | 555399 | Human CD14 Protein | 1:32 | Allophycocyanin (APC) |
| Alexa Fluor® 488 Mouse Anti-Stat1 (pY701) | Monoclonal | Mouse | BD Biosciences | 612596 | Phosphorylated Human Stat1 Peptide | 1:17 | Alexa Fluor® 488 |
| Phospho-STAT2 (Y689) Fluorescein-conjugated Antibody | Polyclonal | Rabbit | R&D Systems | IC2890F | Phosphopeptide containing the human STAT2 Y689 site | 1:20 | Fluorescein (FITC) |
| Human IFN-alpha/beta R1 Fluorescein-conjugated Antibody | Monoclonal | Mouse | R&D Systems | FAB245F | Recombinant human IFN-alpha / beta R1Lys28-Lys436 | 1:10 | Fluorescein(FITC) |
| PE Conjugated Anti-Human IFNAR2 Antibody | Monoclonal | Mouse | PBL Assay Science | 21385-3 | Human interferon alpha/beta receptor chain 2 | 1:20 | R-Phycoerythrin(PE) |

**S1Table**. Detailed information of the antibodies used in flow cytometry.
